# The importance of multimodal CT examination in stroke mimics diagnosis: design of prospective observational multicentre study

**DOI:** 10.3389/fneur.2024.1365986

**Published:** 2024-06-04

**Authors:** Kateřina Dvorníková, Veronika Kunešová, Marcela Ely, Svatopluk Ostrý, Martin Čábal, Martin Reiser, Linda Machová, Markéta Pavlínová, Adéla Kondé, Pavel Eliáš, Tomáš Jonszta, Jaroslav Havelka, Ondrej Volný, Michal Bar

**Affiliations:** ^1^Department of Neurology, University Hospital Ostrava, Ostrava, Czechia; ^2^Department of Imaging Methods, Faculty of Medicine, Ostrava of University, Ostrava, Czechia; ^3^Cerebrovascular research program, International Clinical Research Center, Brno, Czechia; ^4^Hospital in České Budejovice A.S., České Budějovice, Czechia; ^5^Department of the Deputy Director for Science, Research and Education, University Hospital in Ostrava, Ostrava, Czechia; ^6^Department of Applied Mathematics, Faculty of Electrical Engineering and Computer Science, VSB – Technical University of Ostrava, Ostrava, Czechia

**Keywords:** CT perfusion, multimodal CT, stroke, stroke mimics, MRI

## Abstract

**Introduction:**

Non-contrast computed tomography (CT) and CT angiography are the gold standard in neuroimaging diagnostics in the case of suspected stroke. CT perfusion (CTP) may play an important role in the diagnosis of stroke mimics (SM), but currently, it is not a standard part of the stroke diagnostic procedure. The project is a multicentre prospective observational clinical research focused on refining the diagnostics of stroke and stroke mimics (SM) in hospital care.

**Aim:**

This study aimed to evaluate the degree of specificity and sensitivity of multimodal CT (NCCT, CTA, and CTP) in the diagnosis of SM versus stroke.

**Methodology:**

In this study, we will include 3,000 patients consecutively admitted to the comprehensive stroke centres with a diagnosis of suspected stroke. On the basis of clinical parameters and the results of multimodal CT and magnetic resonance imaging (MRI), the diagnosis of stroke and SM will be established. To clarify the significance of the use of the multimodal CT scan, the analysis will include a comparison of the blinded results for each imaging scan performed by radiologists and AI technology and a comparison of the initial and final diagnosis of the enrolled patients. Based on our results, we will compare the economic indicators and costs that would be saved by not providing inadequate treatment to patients with SM.

**Conclusion:**

The expected outcome is to present an optimised diagnostic procedure that results in a faster and more accurate diagnosis, thereby eliminating the risk of inadequate treatment in patients with SM.

**Clinical trial registration:**

clinicaltrials.gov, NCT06045455.

## Introduction

Stroke mimics (SM) present a wide variety of neurological disorders that exhibit symptoms similar to those of a proven cerebrovascular accident (CVA—stroke), despite not being a true stroke. Every year, stroke affects approximately 28,000 people across the entire age spectrum in the Czech Republic, out of which one-third of patients succumb to the damage, some patients remain with a permanent deficit, and only a proportion of these patients recover fully from this disease. To achieve a positive outcome for patients with stroke, it is essential to initiate treatment early, which requires the early recognition of symptoms and the diagnosis of stroke. However, a rapid assessment can lead to the unintentional treatment of patients with SM, which is not indicated, is costly, and can lead to complications. A meta-analysis of data from 62,664 patients indicates that the average prevalence of SM is 24.8% (with an interval of 20–50%). The frequency of SM varies and depends on where the diagnosis is conducted and whether the patients are assessed by a physician at the emergency department or by a neurologist (1, 2).

SM can be divided into two main categories: medical SM and functional SM. Medical SM consist of brain disorders (migraine, seizures, posterior reversible encephalopathy syndrome/PRES, epidural/subdural haematomas, hypertensive encephalopathy, or brain tumours) and systemic diseases (electrolyte dysfunction and metabolic/toxic disorders). The frequency of the individual types of SM varies across different studies. The most recent comprehensive review showed the following frequencies of the different types of SM: peripheral vestibular syndrome 23.2%, toxic/metabolic disorders 13.2%, seizures 13%, migraine 7.8%, syncope 6%, mononeuropathy 5%, space-occupying brain lesions 4%, acute confusion 1.9%, dementia 1.2%, and spinal cord lesions 0.7% (3).

CT perfusion (CTP) identifies the ischaemic brain tissue in patients with suspected stroke even when non-contrast head computed tomography (NCCT) is normal (4, 5). CTP can estimate brain regions with a high probability of irreversible infarction (ischaemic core) and areas at risk of infarction (penumbra). CTP is a functional examination of the brain tissue that characterises the state of brain perfusion and provides information on the current functional state of the microcirculation (6). These facts provide very important prognostic information in the cases of successful and unsuccessful acute recanalisation treatment. CTP allows the selection of suitable candidates for recanalisation treatment after 6 h in the case of mechanical thrombectomy (MT) and after 4.5 h in the case of intravenous thrombolysis (IVT), or if the time of stroke onset is unknown. Recent studies have demonstrated the benefit of MT within 6 h from the onset of stroke in patients with a large ischaemic core. The importance of CTP as an indication for acute recanalisation treatment has decreased, but the prognostic and diagnostic roles of CTP is still essential (7). Perfusion imaging abnormalities have also been reported to be useful in differentiating SM from acute ischaemic stroke and may inform treatment decision-making (8–11).

Exclusion of ischaemic stroke and early detection of SM are crucial in reducing the number of unindicated intravenous thrombolysis (IVT) treatments, which is the first choice of treatment for patients with stroke. In the randomised Norwegian Tenecteplase Stroke Trial (NOR-TEST) study that assessed the effect of tenecteplase in ischaemic strokes, IVT was administered to 18% of patients (tenecteplase vs. alteplase) with a diagnosis other than ischaemic stroke (12). Unindicated administration of IVT can cause possible negative implications for patients (extra or intracerebral haemorrhage, orolingual angioedema, etc.), thereby leading to permanent health and social stigma.

The existing literature predominantly consists of a small quantity of case series concerning SM and have often included only patients with seizures or migraines. In reality, the spectrum of SM is much wider. In this respect, the present project is unique when compared with the current published studies.

## Overall goals of the project

This project aims to optimise hospital diagnostics for stroke and SM symptoms, focusing on the specificity and sensitivity of multimodal brain imaging compared to conventional imaging. The specific goals of the study include evaluating diagnostic outcomes between blinded radiologists/AI technology and clinical diagnosis as well as comparing economic indicators to identify potential savings from avoiding unnecessary treatments.

## Methods

The study employs a prospective multicentre observational design in two Czech comprehensive stroke centres, enrolling an estimated 3,000 patients over 3 years.

The inclusion criteria include acute neurological deficits with symptoms lasting up to 24 h and NIHSS ≥0 points (National Institutes of Health Stroke Scale) at enrolment.

**Figure 1:**
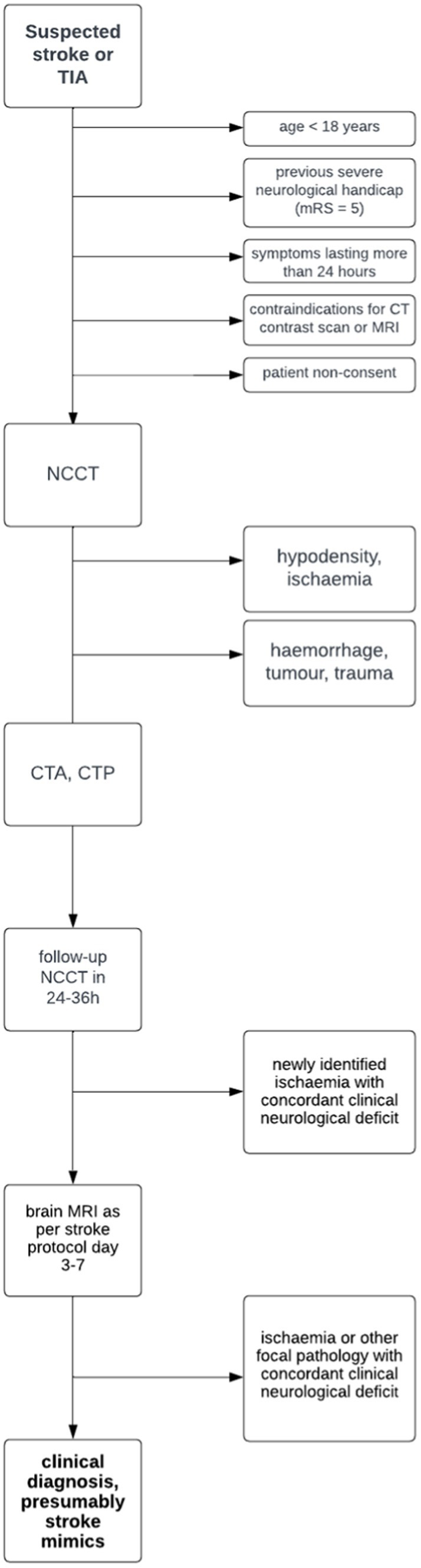
STARD diagram.

The exclusion criteria include age < 18 years, previous severe neurological handicap (mRS = 5), contraindications for CT contrast scan or MRI (pregnancy, severe renal failure, allergy to contrast material, or claustrophobia), and patient non-consent. For the trial flow, see the STARD diagram (Figure 1).

### Monitored parameters

The following data will be collected and evaluated in Table 1.

**Table 1:** Following data will be collected and evaluated.

Patient history	
Age Sex Comorbidities Long-term medication	Hypertension, diabetes mellitus, heart disease, arrhythmia, hyperlipidemia, mental disorders, cephalgia, migraine, epilepsy, oncological disease, cognitive deficit antithrombotic medication, hormonal anticonception
Hospitalisation data	
Date and time of stroke/stroke mimics Date of hospital admission Hospitalisation, duration	standard, ICU
Systolic/diastolic blood pressure Laboratory parameters-INR, glycemia, Increase in liver tests, kidney failure, drugs in the blood	
Clinical data	
mRS NIHSS Neurological deficit Type of treatment Multimodal brain CT scan on admission Follow-up NCCT Brain MRI results Final diagnosis	Pre-morbid, at discharge on admission, 24 h after the admission to hospital NCCT, CTA, CTP during 24–36 h after the admission in indicated patients stroke, stroke mimics
Follow-up visit	
mRS value Recurrence of symptoms Clinical condition	

### Stroke protocol

#### Multimodal CT

All patients with acute neurological deficits (stroke, transient ischaemic stroke (TIA), or SM) will undergo neuroimaging diagnostic procedures as per the stroke protocol, i.e., initial multimodal brain CT (NCCT, CTP, and computed tomography angiography (CTA)), followed by follow-up NCCT within 24–36 h.

The basic CTP parameters define perfusion maps, which express the mean transit time, cerebral blood flow, and cerebral blood volume. Currently, the maps defining the so-called maximum time (Tmax) are also essential. The AI software package allows us to determine the extent of irreversible ischaemia (decrease in CBF below 30% of the reference bilateral value) and potentially the tissue that can be rescued, i.e., penumbra (prolongation of Tmax over 6 s).

Type of CT device—CT Force Siemens.

Stroke protocol: Unenhanced brain: 120 kV (CarekV), 300 mAs (CareDose), collimation 128 × 0.6 mm rotation time 0.5 s Recon: slice 0.75 mm, 5 mm, kernel Hr 40.

CTP: 70 kV, 200 mAs, collimation 48×1.2 mm, brain coverage-4D range: 114 mm.

Cycle time 1.5 s exam time 45 s rotation time 0.25 s contrast medium—40 mL intravenous.

CTA: 120 kV (CarekV), 84 mAs (Care Dose), collimation 128 × 0.6 mm.

Recon: slice 0.5 mm, 3 mm, kernel Hr 36, contrast medium—50 mL intravenous.

#### MRI examination

All patients without a confirmed concordant hypoperfusion or cerebral ischaemia on their previous CT scans will undergo an MRI examination between the third and seventh day after admission to hospital to confirm the diagnosis of SM.

The MRI examination will be performed using the Siemens Prisma, 3.0 T scanner (Siemens, Erlangen, Germany). The imaging protocol includes:

- localisers;
- diffusion weighted imaging (DWI) with b-factor values 0, 400, and 800 s/mm2, calculated b-value of 1,000 s/mm2. ST 3.0 mm, GAP 0.6 mm, TR 2900 ms, TE 60 ms, FOV 256 mm, BW 868 Hz/Px, number of slices 40, TA 2:09;
- reconstructed maps of an apparent diffusion coefficient (ADC maps);
- 3D FLAIR sequence. The examination will be performed in the sagittal plane with 1 mm MPR axial and coronal reconstructions. TR 5500 ms, TE 383 ms, ST 1 mm, number of slices 160, GAP 0, TI 1800 ms, FA T2 Var. deg., BW 751 Hz/Px, TF 220, TA—acquisition time 3:46. Total examination time 6:12+ shimming time.

Follow-up brain MRI will be performed on the following patients as per the stroke protocol:

1. Patients with a negative initial multimodal CT scan.
2. Patients with discordant CTP results, which means that the location of the hypoperfusion does not correspond with the neurological symptoms, or patients with CTP technical artefacts.
3. Patients with a negative follow-up brain NCCT.
4. Patients with an NIHSS score of zero upon admission to the hospital and 24 h after the onset of TIA.

### Analyse of CT and MRI examination

The results of NCCT, CTA, and CTP will be evaluated by a blinded radiologist against the clinical diagnosis and will then be compared with the multimodal CT examination evaluated by AI. The concordance of a positive CTP finding in relation to the clinic will be assessed by a specialist stroke neurologist. The AI software (e-Stroke, Brainomix, Oxford) provides a fully automated scoring tool for analysing CT and MRI examinations. The software calculates e-ASPECT and the volume of ischaemia; it analyses CTP and the values of collateral circulation. Furthermore, it can determine the area of vascular occlusion and the size of ischaemia on MRI. In this respect, Brainomix e-STROKE software is a unique and irreplaceable tool.

### Determination of the definitive diagnosis

Two neurologists specialised in treating and diagnosing stroke will confirm the diagnosis independently.

The following patients will be diagnosed with an ischaemic stroke:

- Patients with a finding of hypodensity corresponding with subacute ischaemia on the initial brain CT examination.
- Patients with a new subacute ischaemic lesion on the follow-up NCCT scan within 36 h from the onset of the neurological deficit.
- Patients with an ischaemic brain lesion on their MRI scan within 3–7 days from the onset of the neurological deficit.

The diagnosis of SM will be determined by the following parameters:

- organic lesion of aetiology other than ischaemic stroke on the NCCT scan,
- negative CTP, discordant CTP, and negative control brain MRI,
- two neurologists and two radiologists specialised in treating and diagnosing stroke will confirm the diagnosis of SM independently, considering the correlation of imaging and clinical data (see clinical data checklist). The radiologists will also assess other thresholds during the CTP examination than the classically established ones (e.g., T max less than 6 s). Both stroke consultants will assess the final diagnosis for the whole group with and without knowledge of the CTP result.

### Follow-up visit

Telephone or outpatient appointment within 90 days from the onset of the neurological deficit. During this visit, the patient’s mRS value, recurrence of symptoms, and clinical condition should be evaluated.

### Quality of life (QoL) evaluation and pharmacoeconomic analysis

We have incorporated a quality of life (QoL) evaluation framework into our study to demonstrate our commitment to a holistic approach to patient care and to enhance the understanding of the benefits of accurate diagnosis beyond the immediate clinical outcome. The QoL framework in our study includes both the Modified Rankin Scale (mRS) and the quality-adjusted life years (QALY) metrics (13).

The mRS is known for its direct relevance to stroke outcomes and its ability to measure changes in functional status. Furthermore, it is simple and easy-to-use tool that is widely accepted and validated in stroke research. In the context of pharmacoeconomic analysis in our study, the mRS provides a quantifiable outcome measure that can be related to QALYs and other economic evaluations. QALYs are commonly used in health economics. Our study aims to highlight how an accurate diagnosis can lead to a better patient outcome and potentially lower healthcare costs over time (14, 15).

### Statistic analysis

A minimum of 718 patients is required to demonstrate significant differences between diagnostic approaches. The analysis involves appropriate statistical tests and logistic regression to assess factors influencing clinical diagnosis. It is expected that the total number of enrolled patients will be 3,000 in order to ensure the availability of all established parameters and, particularly, the relevance of the data obtained. The sample size was determined using the proportional data from the pilot project (*p* = 0.34 for the observation group and a desired effect of at least 10%), 5% significance level, and 80% power.

Numerical variables are presented as medians and ranges or interquartile ranges. Categorical variables are presented as absolute and relative frequencies. The association between demographic, clinical, or radiographic variables and the clinical diagnosis (stroke and stroke mimic) is analysed using appropriate statistical tests—Mann–Whitney test, chi-squared test of independence for contingency tables, or other statistical tests, if necessary. Eventually, logistic regression can be used for analysing the effect of multiple factors on the clinical diagnosis.

The diagnostic accuracy of applied diagnostic methods will be evaluated using common measurements, including accuracy, sensitivity, specificity, positive predictive value, and negative predictive value. The characteristics will be reported with their confidence intervals. The significance level is set to 5%, and statistical analyses will be performed using appropriate statistical software (R, STATA) with maximum available data.

The pharmacoeconomic analysis will include a health technology assessment (HTA) to calculate the potential value of innovation at an early stage, analysing not only the QoL of patients but also the costs associated with the treatment of stroke and SM. The calculation of costs is based on the average cost of thrombolysis and its associated treatment, representing the incremental cost (IC). Economic evaluation is organised as a test of dominance of improved results (meaning fewer complications associated with the administration of thrombolysis) and lower costs.

To calculate the overall benefit, the value of the QoL and the specific parameter of the quality-adjusted life years (QALY) set for the Czech Republic will be used.

The results will be reported as expected ICs and effects (E) expressed in quality-adjusted years of life (QALY).

#### Pilot project

The design of the proposed research project is based on the pilot analysis that was carried out in the Comprehensive Stroke Centre in the Hospital in Ceske Budejovice, where data from a total of 109 patients with suspected stroke were evaluated. These patients underwent multimodal CT examinations (NCCT, multiphase CTA, and CTP) as per the stroke protocol. MRI examination DWI/FLAIR mismatch was selectively indicated as determined by the clinical context.

Based on the performed examinations, stroke was detected in 72 cases, and SM were detected in 37 cases (34%) (see Tables 2, 3).

**Table 2:** Demographic data of patients in a pilot project.

Suspected stroke		109 patients	
Stroke	66%	Stroke mimics	34%
Male	53%	Male	51%
Female	47%	Female	49%
Age (average value)	71.1 ± 15.5	Age (average’)	67.9 ± 15.5

**Table 3:** Detection of stroke mimics.

Stroke mimics	37	34.0%
Metabolic and/or cardiovascular disorder	7	6.4%
Seizures	5	4.6%
Functional disorder	3	2.8%
Trauma	3	2.8%
Neuropsychiatric manifestations	3	2.8%
Drug intoxication	2	1.8%
Bell’s palsy	1	0.9%
Vestibular syndrome	1	0.9%
Migraine	1	0.9%
Spondylogenic symptom	1	0.9%
Inconclusive or unexplained cause	10	9.2%

Seven patients (6.4%) had symptoms caused by a metabolic disorder and/or a cardiovascular disorder; five patients (4.6%) had symptoms of seizures; and three patients in each group (2.8%) had symptoms of functional disorder, trauma, and neuropsychiatric manifestations (see Table 2). The SM diagnosis was determined in the cases of drug intoxication in two patients (1.8%) and Bell’s palsy, vestibular syndrome, migraine, and spondylogenic symptoms in one patient. An inconclusive or unexplained cause of health complications was found in a total of 10 (9.2%) cases.

## Discussion

This project aims to clarify stroke and SM symptoms in hospital care, potentially modifying care provision. It could substantiate international publications and validate the role of multimodal CT in stroke and SM diagnosis.

It is common knowledge that the examination with the highest sensitivity and specificity for the diagnosis of ischaemic stroke is the MR examination of the brain (16). MR examination has numerous contraindications, and as part of an acute diagnosis, stroke is not routinely used. On the other hand, multimodal CT examinations (including non-contrast head CT (NCCT), head and neck CT angiography (CTA), and CTP) may play a significant role in providing accurate information about brain pathology in a short period of time. NCCT (17) and head and neck CTA, which are commonly used for the detection of large vessel occlusion, remain the gold standard in neuroimaging diagnostics in the case of suspected stroke, whilst CTP may play an important role in the diagnosis of SM. However, CTP is currently not a standard part of the diagnostic procedure in most stroke centres. Most patients with SM exhibited discordant clinical findings when compared to CTP imaging. Normal or entirely discordant Tmax of 6 s abnormalities are more common in patients with SM. Therefore, CTP could be crucial when deciding on the use of intravenous thrombolytic therapy (4). The sensitivity of acute NCCT examination for the diagnosis of ischaemic stroke is low (33%) (5).

The outcome of this project is based on the evaluation that the combination of NCCT, CTA, and CTP is superior to NCCT alone or the combination of NCCT and CTA in the diagnosis of SM in patients with acute neurological deficits. The final diagnosis of SM will be determined based on a unique combination of knowledge of clinical data and multimodal CT and MRI results.

## Conclusion

The project’s anticipated outcome is an optimised diagnostic procedure, reducing the risk of inadequate treatment for suspected stroke patients. The applied research may yield health and socioeconomic benefits, improving healthcare provision and reducing costs.

## Ethics statement

The studies involving humans were approved by Ethics Committee at the University Hospital in Ostrava, No. 503/2022, 23.6.2022; Ethics Committee at the Hospital in České Budějovice, No. 109/22, 24.6.2022. The studies were conducted in accordance with the local legislation and institutional requirements. The participants provided their written informed consent to participate in this study.

## Author contributions

KD: Investigation, Methodology, Writing – original draft, Writing – review & editing. VK: Conceptualization, Funding acquisition, Project administration, Resources, Writing – original draft, Writing – review & editing. ME: Conceptualization, Data curation, Software, Writing – review & editing. SO: Conceptualization, Investigation, Methodology, Supervision, Visualization, Writing – review & editing. MČ: Data curation, Investigation, Methodology, Writing – review & editing. MR: Investigation, Methodology, Writing – review & editing, Data curation. LM: Data curation, Investigation, Methodology, Writing – review & editing. MP: Data curation, Investigation, Methodology, Writing – review & editing. AK: Formal analysis, Methodology, Project administration, Writing – review & editing. PE: Conceptualization, Formal analysis, Methodology, Software, Supervision, Writing – review & editing. TJ: Data curation, Investigation, Methodology, Software, Writing – review & editing. JH: Data curation, Investigation, Methodology, Software, Writing – review & editing. OV: Formal analysis, Investigation, Methodology, Supervision, Visualization, Writing – review & editing. MB: Conceptualization, Data curation, Formal analysis, Funding acquisition, Investigation, Methodology, Project administration, Resources, Software, Supervision, Validation, Visualization, Writing – original draft, Writing – review & editing.
