# Supplementary material for: The importance of multimodal CT examination in stroke mimics diagnosis: design of prospective observational multicentre study
Source: Front Neurol. 2024 Jun 4;15:1365986. doi: 10.3389/fneur.2024.1365986 (PMC11184135; doi:10.3389/fneur.2024.1365986)
Supplement: Supplementary file 1 [file Data_Sheet_1.PDF]

CHECKLIST		
	**STROKE**	**STROKE MIMICS**
**age and sex**	older age (male = female)	younger age (female > males)
**level of consciousness**	awake	altered level of consciousness
**symptoms severity**	severe at onset	fluctuations in severity are common
**vascular risk factors – DM, arterial hypertension, hyperlipidemia**	yes	no
**migraine**	no	yes
**seizures**	no	yes
**vascular territory – signs and syndromes**	vascular syndromes	no vascular distribution
**blood pressures at presentation**	increase blood pressure at onset is common	blood pressure usually not increased
**signs and symptoms**	weakness (pyramidal distribution), aphasia and visual filed defects	sensory, vertigo (dizziness)
**imaging**	imaging shows ischaemic lesions	no new ischemic lesion
**psychiatric history**	no	yes
